# Simulation and measurement of optimized microwave reflectivity for carbon nanotube absorber by controlling electromagnetic factors

**DOI:** 10.1038/s41598-017-00372-9

**Published:** 2017-03-28

**Authors:** Danfeng Zhang, Zhifeng Hao, Yannan Qian, Yinxin Huang, Bizeng, Zhenda Yang, Wu Qibai

**Affiliations:** 1 Guangdong University of Technology, Guangzhou, 510006 China; 2 Foshan University, Foshan, 528000 China

## Abstract

Heat-treatments may change the defect and surface organic groups of carbon nanotubes (CNTs), and lead to significant changes in the microwave electromagnetic parameter of CNTs. In this paper, the effect of heat-treatment time and temperature on the complex dielectric constant and permeability as well as the microwave reflectivity of CNTs was investigated. The experimental results indicated that the microwave absorption property of CNTs arises mainly from the high permittivity and consequent dielectric loss. Moreover, the heat-treatment resulted in increased dielectric constant of CNTs and significant improvement of the microwave absorption at frequency values of 2–18 GHz. The microwave reflectivity of CNT composites with a coating thickness of 3 mm was simulated by using the electromagnetic parameters. The absorption peak of CNTs treated at 700 °C had an amplitude of R = −48 dB, which occurred at 9 GHz. Below −10 dB, the composites treated at 900 °C had a bandwidth of 7 GHz. The position of the absorption peak concurred with the measured results. The results indicated that the microwave-absorption properties can be modified by adjusting heat-treatment temperature and time.

## Introduction

In recent years, carbon-based nano-materials, such as carbon nanotubes (CNTs), carbon nanoparticles, and carbon onion have been considered as electromagnetic shielding and absorption materials with wide bandwidth, high absorption, tunable electromagnetic properties, and high reliability^1–9^. A good absorption material must effectively absorb and attenuate the incident electromagnetic wave. The design of electromagnetic absorption materials and structure is based on the transmission theory of electromagnetic waves in a medium. In addition, large complex permittivity and permeability of the materials are required for effective absorption, and impedance matching of the materials should be considered. Careful selection and preparation of an impedance matching layer with excellent complex permittivity are essential for dielectric loss-absorbing materials. The frequency characteristics of absorbing materials are determined by the thickness of the coating, the correlation between the concentration and frequency of the absorber, and weight proportion of the materials. CNTs, as a representative one-dimensional nanomaterial, have extraordinary electromagnetic wave-absorption properties and, hence, are considered as the promising excellent absorbent materials that exhibit good compatibility and possess excellent light quality and broad absorbing band width. Abundant dangling bonds in the surface of CNTs could enhance the superficial polarization and the dielectric loss. Moreover, the high specific surface area could result in the multiple scattering. These factors play an important role in the CNTs’ absorption properties, such as the high absorption and wide-frequency band. CNTs have a series of comprehensive performances such as thermal and electrical conductivity, high strength, excellent corrosion resistance, thermal shock resistance, and high-temperature resistance and, hence, act as a high-temperature microwave absorber^8^. The low density of CNTs is favorable for obtaining light-weight composites. Therefore, CNTs have become one of the most important research topics in the new generation of “thin, light, wide, strong” absorbing materials.

The permittivity and permeability behaviors of composites fabricated from multi-walled CNTs with magnetic impurity Ni and wax had been studied at frequencies of 3–18 GHz^9^. The 1.5-mm-thick composites exhibited excellent microwave absorption of the reflection loss (−20 dB). Kim^10^ fabricated hybrids of oxidized multi-walled CNTs and silver powder in a polyurethane matrix for electromagnetic interference shielding. Qi *et al*.^11^ synthesized bamboo-like multi-walled CNTs (BCNTs), which exhibited a reflection loss of −13.32 dB at 14.33 GHz, and a frequency bandwidth of >3 GHz was as lower than the reflection loss of −10 dB. Liu *et al*.^12^ found that the microwave absorption can be optimized at a given concentration and the absorption peak shifts with CNT content. Hou *et al*.^13^ suggested that a bilayer structure could increase the electromagnetic absorption of the CNT composite. Zhai *et al*.^14^ investigated microwave-absorbing materials are composed of hydrogenated butadiene-acrylonitrile rubber (HNBR), carbon fiber (CF), carbon black (CB) and BCNTs. The reflection losses of −49.3 dB, −13.1 dB and −7.1 dB occurred at HNBR/BCNTs, HNBR/CF and HNBR/CB weight ratios of 100/10, 100/15 and 100/30, respectively. Despite excellent absorption properties, the mechanical properties of HNBR/BCNTs composite materials were also improved^15^.

Heat treatments will change the defects and surface organic groups, such as the hydroxyl and carboxyl of the CNTs. The effect of CNT content on the microwave dielectric performance of composite materials has been extensively investigated^12, 14, 16–18^. However, the dependence of the absorption intensity and frequency on the heat-treatment condition of CNTs has scarcely been quantified. Therefore, in this work, CNTs are heat-treated at different temperatures and times, and the corresponding pre- and post-treatment microwave dielectric properties are compared. The relevant electromagnetic parameters of the CNTs are also measured. Furthermore, the reflectivity of the CNT absorber coating is simulated based on the transmission line theory of an electromagnetic wave in a medium, and this reflectivity is compared with the experimental results.

## Experimental

### Calculating the reflectivity of absorbing wave coating by using the transmission line theory

Electromagnetic waves incident from free space into the medium interface may partially reflect on the surface and partially enter the interior of layer or multilayer absorbent materials of a certain thickness with the metallic substrates. Attenuation of electromagnetic waves or the electromagnetic-wave absorption of materials results from multiple repeated reflections between the material surface and the metal surface in the back. The passage of electromagnetic waves through the coating surface and N layer dielectric to the metallic base plate is considered as a multi-node lossy transmission line segment of a terminal short-circuit terminal. The characteristic impedance of the electromagnetic wave between each internode is considered, rather than the times of reflection and refraction interlayer and the degree of absorption. The total characteristic impedance of the entire coating is obtained via the recurrence relation, where the entire coating is considered as a monolayer during calculation of the reflectivity (absorption). This process is referred to as the transmission line method, which is used to calculate the reflectivity of the microwave-absorption coatings.

Here, the coating on the metallic substrate is considered during vertical incidence of the electromagnetic wave. Using the transmission line method, the reflectivity R (absorption, dB) resulting from incidence of an electromagnetic wave on an *N* layer coating can be determined as follows:

1 $$R=20\,\mathrm{lg}|\frac{{Z}_{n}-{Z}_{a}}{{Z}_{n}+{Z}_{a}}|$$

where *Z*_*a*_ is the intrinsic impedance of air, and *Z*_*n*_ is the input impedance of the interface between the *N* layer material and the air, and the reflectivity unit is decibels (dB). Eq. (1) can be normalized as follows:

2 $$R=20\,\mathrm{lg}|\frac{{Z}_{n}-1}{{Z}_{n}+1}|$$

where *Z*_*n*_ is the input impedance of the normalized *N* layer material. The recursion relation of each layer impedance is given as:

3 $${Z}_{n}=\frac{{Z}_{n-1}+{\eta }_{n}th({\gamma }_{n}{d}_{n})}{{\eta }_{n}+{Z}_{n-1}th({\gamma }_{n}{d}_{n})}$$

4 $${\eta }_{n}=\sqrt{{\mu }_{rn}/{\varepsilon }_{rn}}$$

5 $${\gamma }_{n}=j2\pi f/c\sqrt{{\mu }_{rn}{\varepsilon }_{rn}}$$

where *c*: velocity of light, *f*: frequency of the electromagnetic wave, *μ*_*m*_ and *ε*_*m*_: complex permeability and permittivity of the *N* layer material, respectively, *d*_*n*_: thickness of the *N* layer material, and *th*: hyperbolic tangent function. The substrate is metallic (*Z*_*0*_ = 0) and, hence, the overall impedance (from the first to the *N* layer coating) can be determined from the recursion formula. In addition, the reflectivity R(*N*) is calculated based on the obtained characteristic impedance of the surface coating (*N* layer).

When the reflectivity of the absorber coating is calculated via the transmission line method, the characteristic impedance of the coating is decided by the complex permittivity ε (or *ε*_*r*_) and permeability *μ* (or *μ*_*r*_), which is used to represent the electromagnetic property of the absorber. The permittivity and permeability under an alternating magnetic field are expressed as *ε* = *ε′* + *jε″* and *μ* = *μ′* + *jμ″* (in plural form). Considering the effect of the medium on electromagnetic-wave absorption, ε″ and *μ*″ > *ε′* and *μ′* is preferable. Impedance matching of the materials is also considered, i.e., the reflection of electromagnetic wave on the incidence interface should be reduced, and the absorption entered the medium of electromagnetic should be enhanced. Therefore, the optimal value of the electromagnetic parameter is confirmed via simulation, and the optimized reflectivity (absorption) of the absorber coating is subsequently obtained.

### Preparation and measurement of nanotubes coating samples

**Figure 1:**
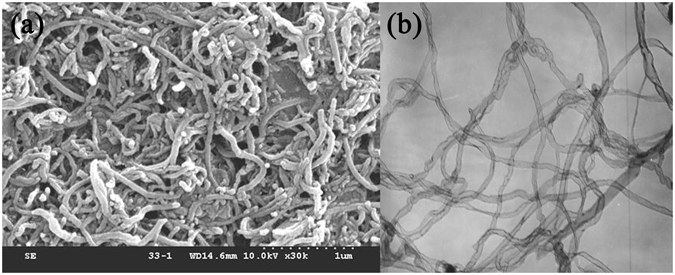
Morphology of the CNT samples heat-treated at 700 °C: (**a**) SEM image and (**b**) TEM image.

A certain amount of CNTs was weighed, and a mixture of sulfuric acid and nitric acid was prepared in a volume ratio of 1:2. The mixed acid and CNTs were homogeneously mixed in a drying flask, heated to 155 °C, and refluxed for 5 h. The mixture was then filtered (i.e., to filter the mixed acid), washed with deionized water, and further filtered until a pH of 7 was obtained for the solution. After low-temperature drying under protective atmosphere, the CNTs were heat-treated at 700 °C and 900 °C, respectively, for 1, 2, 3, and 4 h. Figure 1 shows the scanning electron microscope (SEM) and transmission electron microscope (TEM) photos of carbon tubes treated with the mixed acid and then heat-treated at 700 °C. As the photos shown, the ports of the carbon tubes are broken and open. These carbon tubes with the pipe diameter of 20–40-nm and the lengths exceeding 1 μm have straight bar and arc morphologies.

The heat-treated CNTs and paraffin were mixed in a weight ratio of 85:15. These mixtures were then placed in the two stick rubber mixing machine, dispersed for 20 min, and pressed into a mold. The resulting circular composites, with inner diameter, outer diameter and length of 3.0 mm, 7.0 mm and 2–3 mm, respectively, meet the requirements of the coaxial measurement. An AV3618 vector network analyzer can be used to determine the electromagnetic parameters of the measured samples at frequencies ranging from 2 to 18 GHz. The electromagnetic parameters of paraffin (*ε′*: ∼2.2 and *μ′*: ∼0.6) are significantly smaller than those of the CNTs. Therefore, the electromagnetic parameters of CNTs are taken as the electromagnetic parameters of the composites. Equation (6) is obtained by substituting the electromagnetic parameters of the heat-treated carbon tube/paraffin composite coating into equation (2). Here, the thickness of the composite coating is set to 2 mm to calculate the microwave reflectivity. In equation (6), R is the reflectivity (dB), *µ*_*r*_ is the relative permeability, *ε*_*r*_ is the relative dielectric constant, d is the coating thickness (mm), *c* is the light velocity of free space, and *f* is frequency.

6 $$R=20{\mathrm{log}}_{10}|\frac{\sqrt{\frac{{\mu }_{r}}{{\varepsilon }_{r}}}\,\tan \,h[j\frac{2\pi f}{c}d\sqrt{{\varepsilon }_{r}{\mu }_{r}}]-1}{\sqrt{\frac{{\mu }_{r}}{{\varepsilon }_{r}}}\,\tan \,h[j\frac{2\pi f}{c}d\sqrt{{\varepsilon }_{r}{\mu }_{r}}]+1}|$$

**Figure 2:**
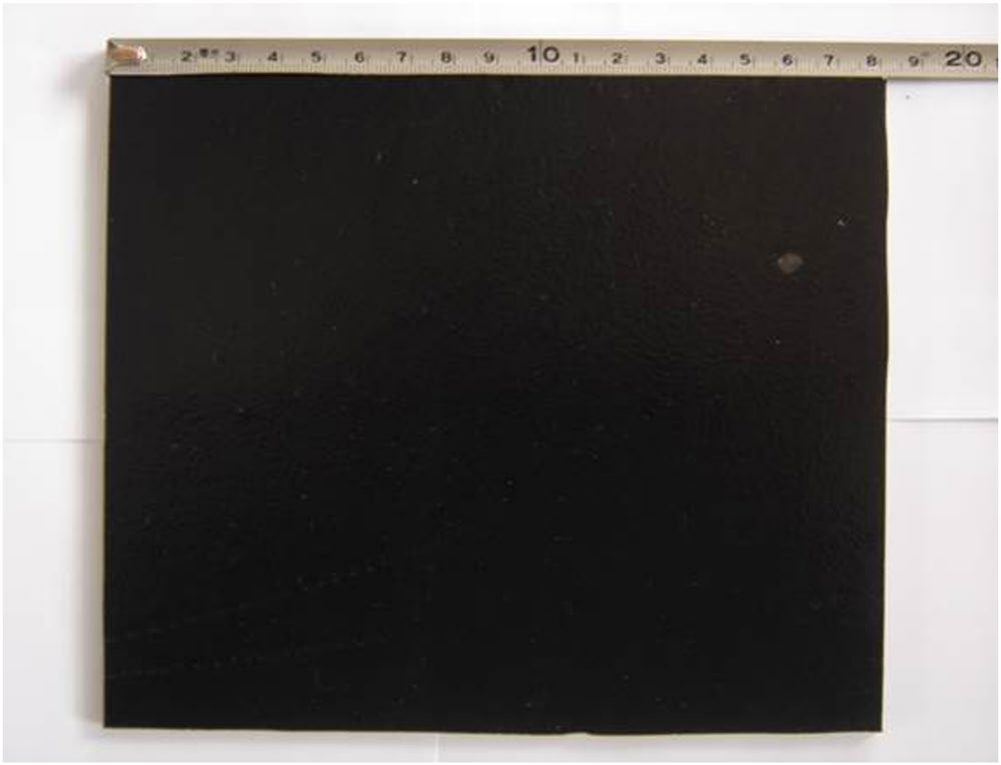
The coating samples were painted onto the 180 mm × 180 mm standard aluminium plate.

**Figure 3:**
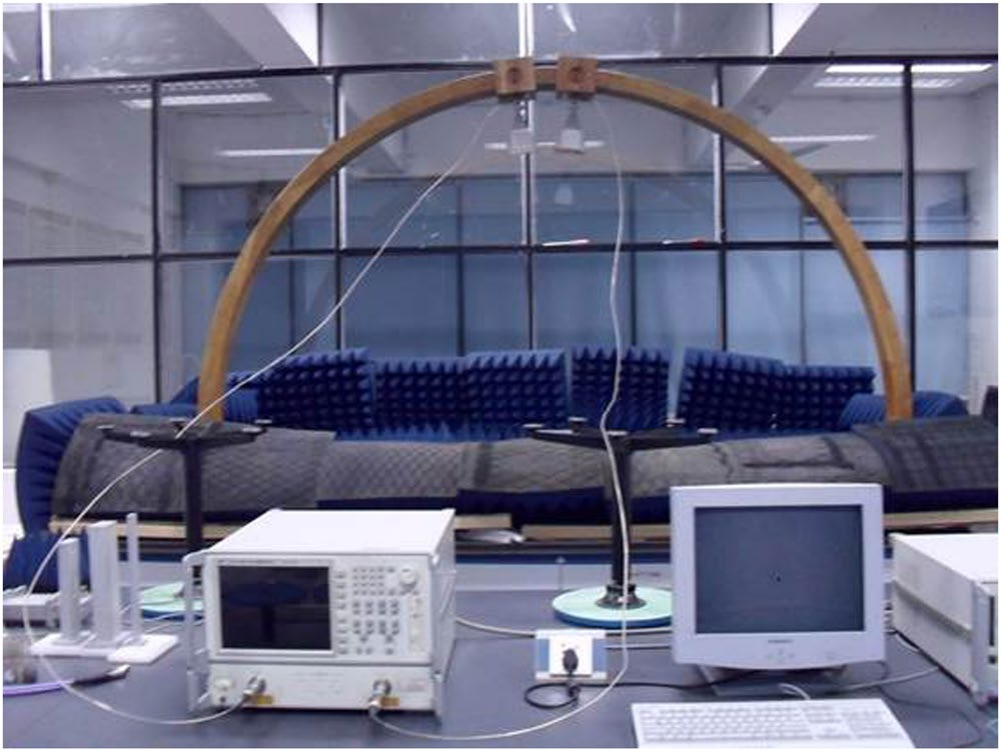
Equipment used to evaluate the practical reflectivity of absorbent materials, via the Arch method.

The measured electromagnetic parameters are imported into Matlab, and R is simulated via the transmission line method for different coating thicknesses. The effect of the heat-treatment temperature and coating thickness on R was also determined. The 180 × 180 mm standard absorption sample plates (see Fig. 2), with a 2-mm-thick coating, are prepared from CNTs and silicone resin composites. The practical reflectivity of the composites is measured via the Arch method (see Fig. 3).

## Results and Discussion

### Relative complex permittivity and permeability of the paraffin-CNTs composites

**Figure 4:**
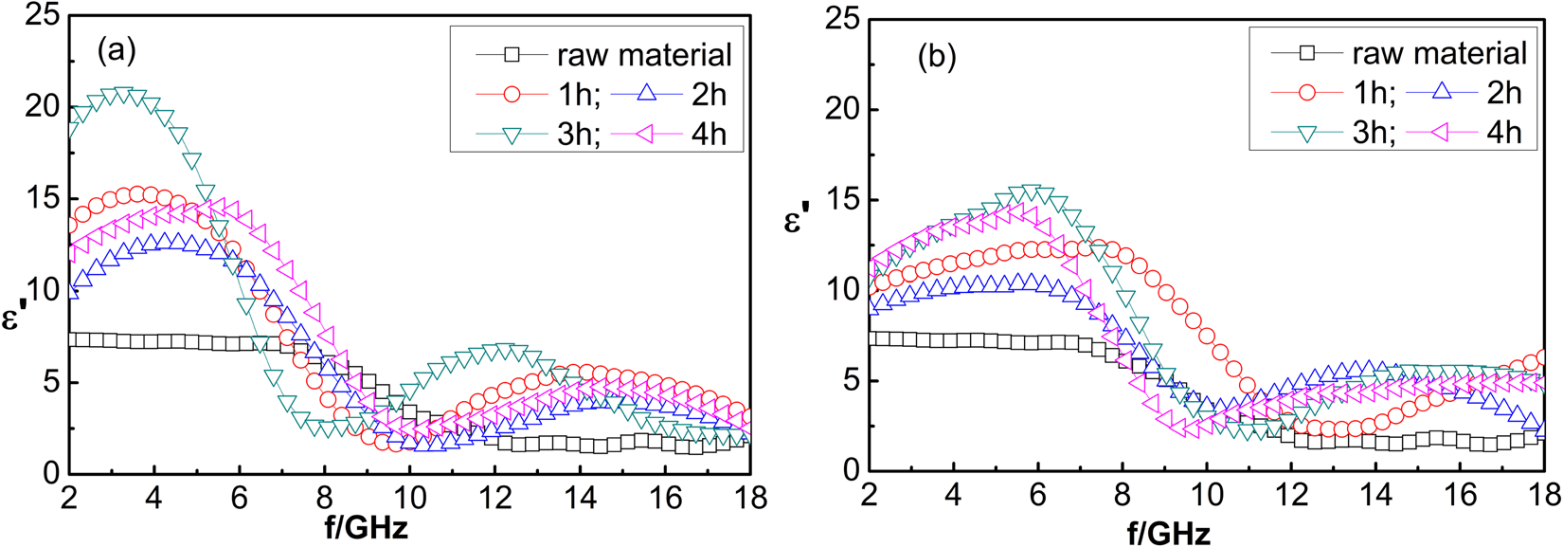
Real part (*ε*′) of the complex dielectric constant of CNT composites heat-treated for 1, 2, 3, and 4 h at temperatures of 700 °C and 900 °C; *ε*′ of the untreated composites is denoted as 0 h.

The real part (*ε′*) of the complex dielectric constant of the CNT composites is measured at microwave frequencies of 2–18 GHz. Figure 4 shows the relationship between the real part of complex dielectric constant and frequency for CNTs heat-treated at temperatures of 700 °C and 900 °C, respectively, for 1, 2, 3 and 4 h. As shown in Fig. 4, the maximum value of real parts (i.e., *ε′* = 21) for heat-treated CNTs occurs in the low-frequency band (2–8 GHz).

In the high-frequency band (9–18 GHz), the heat treatment has only a modest effect on *ε′*, as evidenced by smaller changes in its value compared to those occurring in the low-frequency band. The experimental results indicate that *ε′* may be affected by the heat-treatment temperature and time. Excessively high temperatures (e.g., 900 °C) should reduce the density of defects and groups, leading to a reduction in the number of electric dipoles and, in turn, the dielectric constant of CNTs. Furthermore, excessively long or short heat-treatment time will lead to a decrease in the dielectric constant. Short times are inadequate for complete optimization of the structure of the CNTs, whereas excessive times will lead to a decrease in the number of CNTs and electric dipoles. This will lead to a decrease in the dielectric constant of the CNTs. The maximum *ε′* is obtained at a heat-treatment time of 3 h.

As CNTs are non-magnetic materials, their absorption originates either from polarization or multiple scattering due to the large specific surface area. Compared with single-walled CNTs (SWCNTs), multi-walled CNTs (MWCNTs) have higher defect densities, and therefore higher permittivity, and their microwave-absorption ability arises mainly from dielectric loss. Previous studies have indicated that the defects and impurities in CNTs are influenced by the polarization of the composite materials^19, 20^. The electromagnetic-wave absorption of CNTs arises mainly from electric dipole steering and interfacial polarization^21, 22^ because the increasing density of defects and groups leads to the increasing density of electric dipoles. Under an external electromagnetic field, the defects and dangling bonds in the CNTs would become the polarization center, and the interfacial polarization of the CNTs as well as the dielectric relaxation and dielectric loss increase.

**Figure 5:**
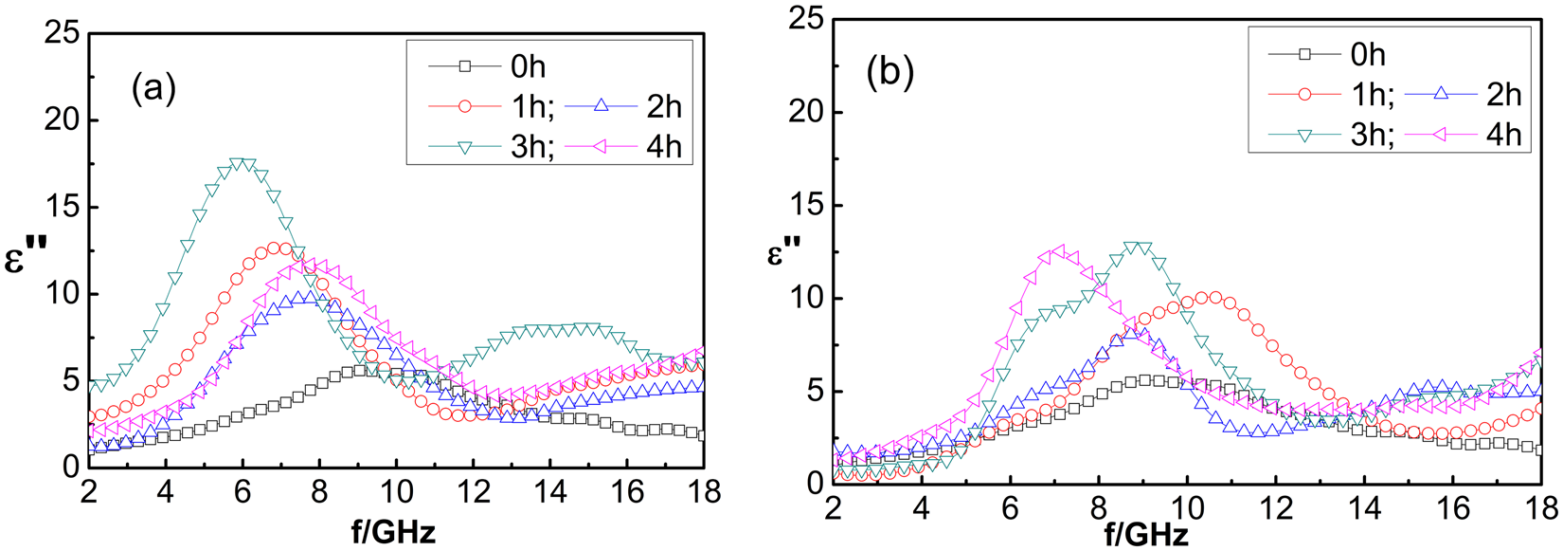
Imaginary part (*ε″*) of the complex dielectric constant of CNT composites heat-treated for 1, 2, 3, and 4 h at temperatures of 700 °C and 900 °C; *ε″* of the untreated composites is denoted as 0 h.

Figure 5 shows the frequency dependence of the imaginary part (*ε″*) of the complex dielectric constant of the CNT composites heat-treated for 1–4 h at 700 °C and 900 °C. As shown in Fig. 5, *ε″* of the heat-treated CNTs exhibits a higher frequency dependence than the *ε″* of the untreated CNTs. The maximum *ε″* of the untreated CNTs (i.e., *ε″* = 5.5) occurs at ∼9 GNz. However, a maximum *ε″* of 17 (i.e., *ε″* = 17) is obtained for the heat-treated CNTs. The maximum *ε″*, associated with each heat-treatment time, occurs in the low-frequency band. The peak in the dielectric loss indicates that the highest absorption occurs at 7–9 GHz. *ε″* increases with increasing heat-treatment time, reaching a maximum value (i.e., 17) at heat-treating time of 3 h, and decreasing thereafter. This indicates that sufficient heat-treatment time is required for structural optimization of CNTs, and insufficient time will lead to an unstable *ε″*. However, excessive heat-treatment time may reduce the number of CNTs, leading to a reduction in the number of electric dipoles. The change in *ε″* is consistent with the change in *ε*′.

Heat treatments may modify the surface structure of CNTs, reduce the defect density, transform the structure, and increase the dielectric constant. The complex dielectric constant obtained via heat treating at 700 °C is larger than that obtained at 900 °C. The shift of the complex dielectric constant to the low-frequency band is conducive for absorption at low frequency. Excessive heat-treatment temperatures may erode carbon tubes, lead to a reduction of electric dipoles, and result in a decrease in the dielectric constant. Therefore, the complex dielectric constant and frequency of CNTs can be tailored by varying the heat-treatment temperature and time.

**Figure 6:**
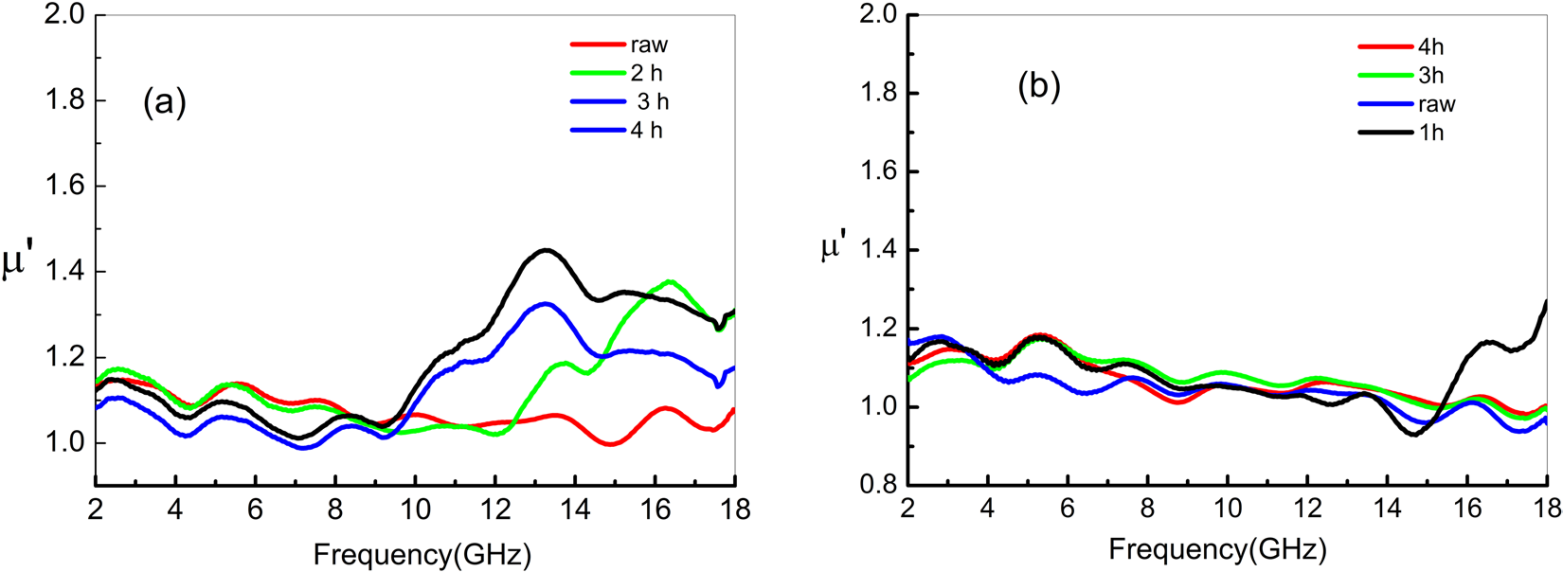
Real part (μ′) of the complex permeability of CNT composites heat-treated for 2, 3, and 4 h at temperatures of 700 °C and 900 °C; μ′ of the untreated composites is denoted as 0 h.

**Figure 7:**
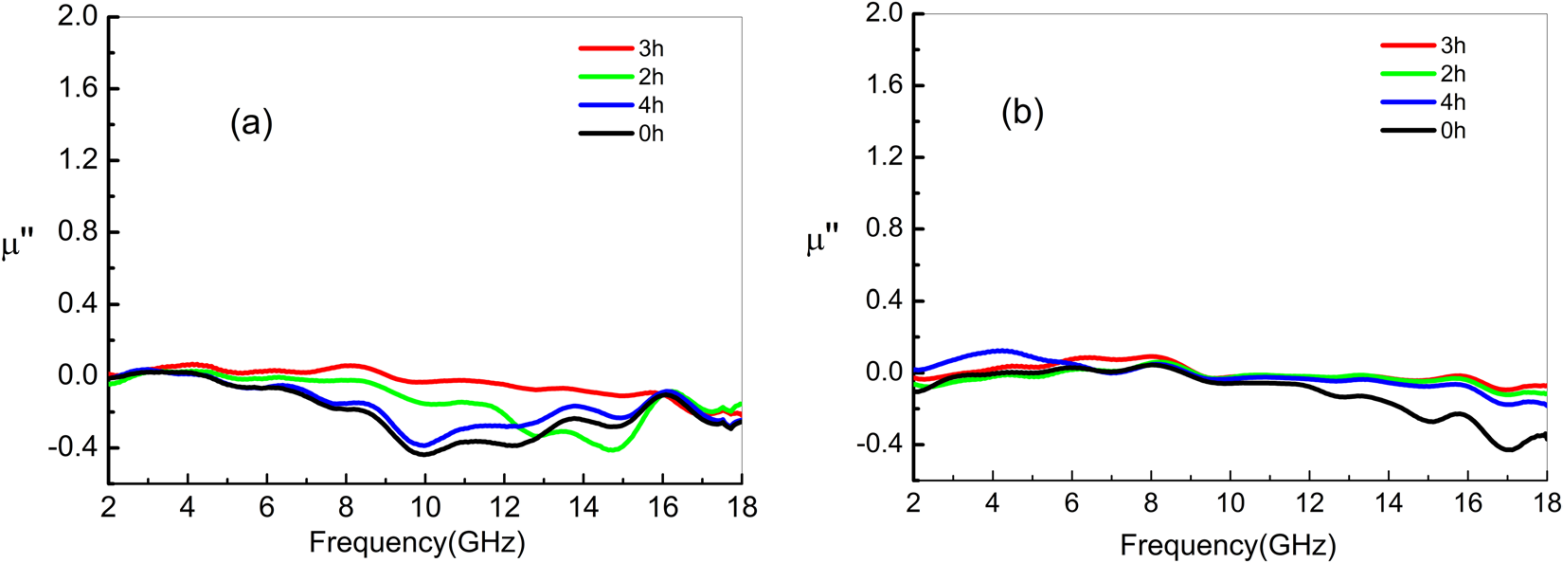
Imaginary part (μ″) of the complex permeability of the CNT composites heat-treated for 2, 3, and 4 h at temperatures of 700 °C and 900 °C; μ″ of the untreated composites is denoted as 0 h.

Figures 6 and 7 show the frequency-dependence of the real (μ′) and imaginary (μ″) parts, respectively, of the permeability of the untreated and (three of the aforementioned) heat-treated CNTs. As Fig. 6 shows, the original untreated CNTs have a μ′ of ∼1.1. The high-temperature treatment has only a slight effect on μ′, which is almost constant at low frequency. At high frequencies, μ′ of the treated CNTs reaches a maximum value of 1.4. Fig. 7 displays the imaginary part (i.e., μ″) of the complex permeability constant for both the treated and untreated composites. As shown in Fig. 7, μ″ of the treated CNTs exhibits the same behavior as the μ″ of the untreated CNTs. This similarity results possibly from the fact that the heat-treatment induced change in the number of electric dipoles affects mainly the dielectric constant rather than the permeability.

### Reflection loss of CNT composites: simulation and measurement

**Figure 8:**
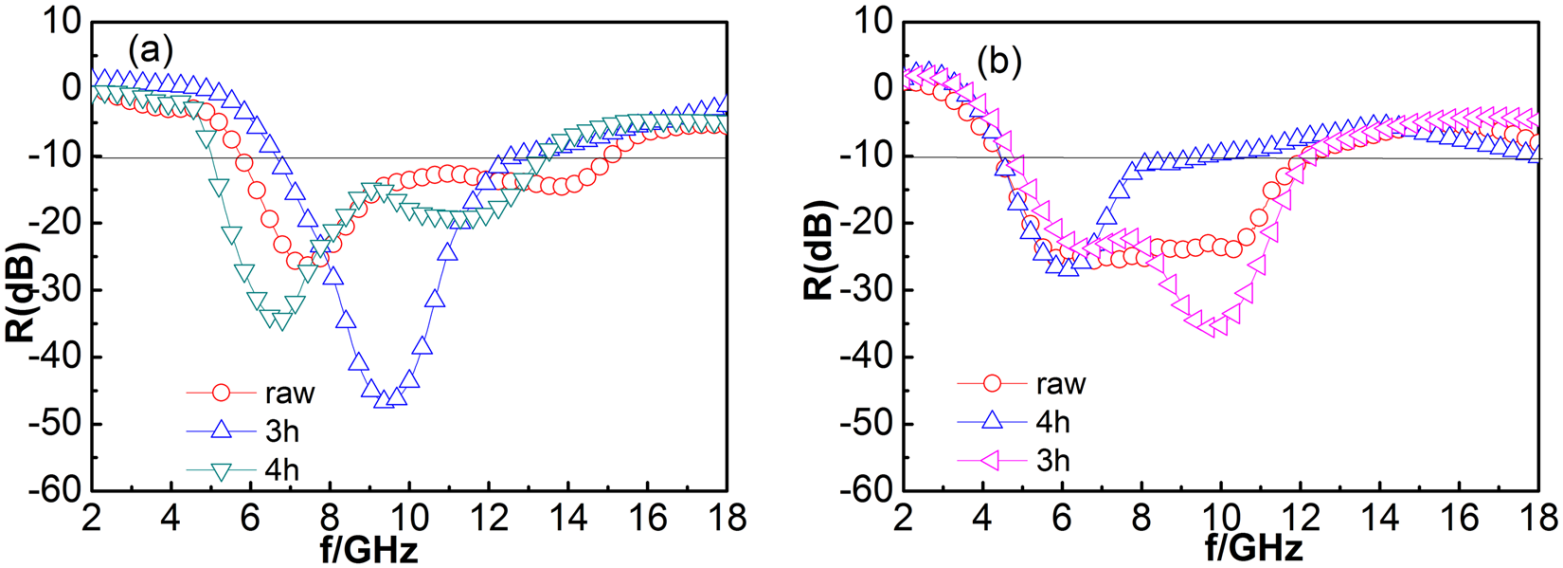
Simulated reflectivity of CNT composites heat-treated for 3 and 4 h at temperatures of 700 °C and 900 °C. The simulated reflectivity of the untreated composites is denoted as 0 h.

The correlation between the electromagnetic parameter and reflectivity of materials indicates that the absorption reflectivity of these materials will increase with increasing real component of the complex dielectric constant. Figure 8 shows the simulated reflectivity of the heat-treated and the untreated composites; these composites consist of 3-mm-thick CNTs, which account for 15 wt.% of the composite. Figure 8(a) shows the reflectivity of CNT composites treated at 700 °C. As the figure shows, the microwave-absorbing reflectivity of the untreated composites occurs at ∼7 GHz, and the maximum absorption-peak reflectivity is −25 dB. Heat treating at the temperature (700 °C) may yield the improved absorption of the CNTs. For example, a peak value of −48 dB and a bandwidth of ∼5 GHz below −10 dB are obtained for the 3 h heat-treated CNT composites. These experimental results are consistent with those obtained, at frequencies of 7–9 GHz, for the imaginary part of the complex dielectric constant. When the heat-treatment time is increased to 4 h, the reflectance absorption peak shifts to low frequency, towards the peak corresponding to the untreated CNTs, and has an amplitude of ∼−37 dB (6.5 GHz). The high temperature of the heat treatment may reduce the internal defects of the CNTs, making the structure more perfect and imparting the intrinsic characteristics (e.g., high dielectric loss) of the CNTs. For the time considered here, the dielectric constant of the carbon tubes increases. However, excessive heat-treatment times would erode the number of carbon tubes and lead to a reduction of electric dipoles, the dielectric constant and, consequently, the reflectivity absorption peak. The observed increase in the absorption peak is, as in the case of previous studies^16–18^, attributed to an increase in the dielectric losses. When the heat-treatment temperature is increased to 900 °C (see Fig. 8(b)), the simulated reflectivity absorption peak of the 3 h-treated composites decreases to −36 dB (at ∼10 GHz). However, a bandwidth of ∼7 GHz, which is expected to increase, occurs below −10 dB. These results indicate that the real and imaginary part of the complex dielectric constant may be modified, and the microwave absorption effect of CNTs further tailored by controlling the heat-treatment temperature and time of the CNTs. This could ensure fulfillment of requirements for various frequency band and bandwidth.

**Figure 9:**
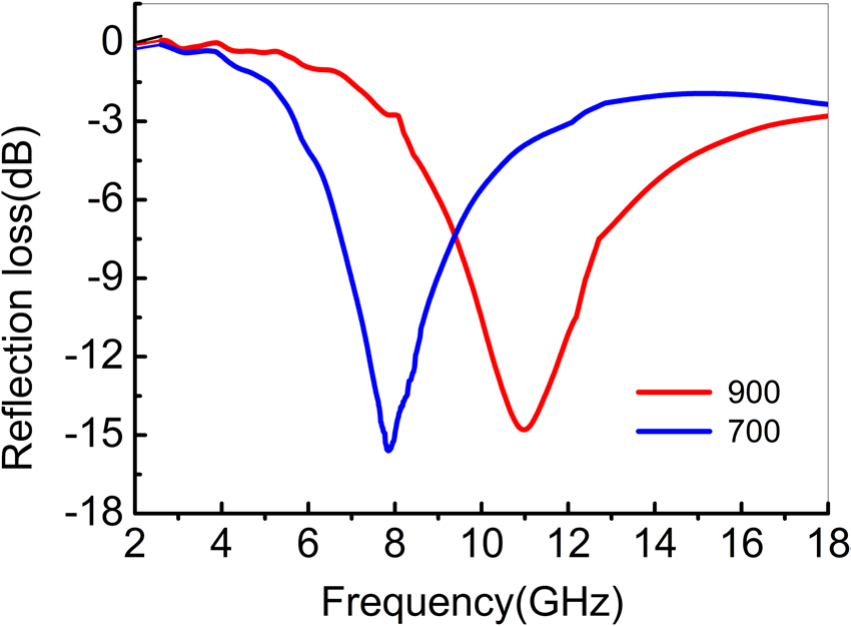
The experimental reflectivity curve of 3-mm-thick CNT composite coatings with a CNT content of 15 wt.%. The composites were heat-treated for 3 h at temperatures of 700 °C and 900 °C.

Figure 9 shows the measured reflectivity curve of a 3-mm-thick composite coating with a CNT content of 15 wt.%; this curve is obtained via the Arch method. As shows in Fig. 9, the absorption peak of the composites treated at 700 °C has a minimum value of −15.2 dB (8 GHz). The corresponding peak of the composite heat-treated at 900 °C has a minimum value of ∼−14.2 dB (11 GHz). This trend is relatively consistent with the simulated results. The theoretical and experimental studies on CNT composites reveal that the interface properties of CNTs are influenced by the heat-treatment process. These properties will ultimately influence the electromagnetic factors and microwave-absorption characteristics. The effect of interfacial polarization was also demonstrated by comparing the microwave-absorption characteristics of CNT/polymer and decorated CNT/polymer composites^23–25^. These observations indicate that strong interfacial polarization is essential for high microwave absorption.

## Conclusion

The effect of heat-treatment temperature and time on the electromagnetic parameters and microwave absorption of CNTs was investigated. The experimental results indicate that heat-treatments can increase the dielectric constant of CNTs and significantly improve their microwave absorption at frequency values of 2–18 GHz. In addition, the microwave reflectivity of the CNT composites, with a coating thickness of 3 mm, are simulated by using the electromagnetic parameters. The absorption peak of CNTs treated at 700 °C has an amplitude of R = −48 dB, which occurs at 9 GHz. Below −10 dB, the composites treated at 900 °C have a bandwidth of 7 GHz. The position of the absorption peak concurs with the measured results. The results indicate that the microwave-absorption properties can be modified by adjusting the heat-treatment temperature and time.
